# Motivation and retrospective appraisal of psychedelic study participation: a qualitative study in healthy volunteers

**DOI:** 10.1007/s00213-025-06772-4

**Published:** 2025-03-26

**Authors:** Laura Ley, Matthias E. Liechti, Anna M. Becker, Isabelle Straumann, Aaron Klaiber, Friederike Holze, Severin B. Vogt, Denis Arikci, Yasmin Schmid

**Affiliations:** 1 Clinical Pharmacology and Toxicology, Department of Biomedicine and Department of Clinical Research, University Hospital Basel and University of Basel, Schanzenstrasse 55, CH-4031 Basel, Switzerland; 2 Department of Pharmaceutical Sciences, University of Basel, Basel, Switzerland

**Keywords:** Psychedelics, Motivation, Appraisal, Healthy volunteers, Lysergic acid diethylamide, Psilocybin, Mescaline, Dimethyltryptamine

## Abstract

**Rationale:**

Little is known about motives of healthy volunteers to participate in psychedelic trials and how they appraise their study experience retrospectively.

**Objectives:**

This paper explored reasons why healthy people register for psychedelic trials, factors that they considered to contribute to either positive or negative study experiences, and under which circumstances they would seek a psychedelic experience again.

**Methods:**

This study used the data of 151 healthy volunteers who had ingested serotonergic psychedelics in one of six randomized, double-blind, placebo-controlled crossover trials at the same research site under similar conditions. The data were analyzed through qualitative content analysis.

**Results:**

The predominant motivations to participate in a trial were interest in psychedelics and an appealing setting. Expectations involved personal development and the occurrence of typical psychedelic effects. Hopes included transformative processes. The setting factors that promoted a positive experience were music and access to nature, whereas the sterile hospital environment was considered bothersome. Most participants valued the trusting relationship with their investigator. The most commonly criticized investigator characteristics were a perceived lack of support and investigator-induced psychological discomfort. Most participants considered their expectations exceeded and would take the study substances again, preferably in a setting in nature with friends.

**Conclusions:**

This paper identified four pivotal factors to be considered for psychedelic study experiences: (1) a secure interpersonal relationship, (2) an aesthetically pleasing environment, (3) access to nature, and (4) the use of music. This analysis reveals subjective views of volunteers in psychedelic Phase-I trials and may improve research standards.

**Supplementary Information:**

The online version contains supplementary material available at 10.1007/s00213-025-06772-4.

## Introduction

The worldwide number of psychedelic clinical trials is continuously growing (Siegel et al. 2021; Kurtz et al. 2022). In recent years, a multitude of Phase-I trials of a broad range of psychedelic substances have been conducted in our laboratory at University Hospital Basel under similar setting conditions (Holze et al. 2022; Ley et al. 2023; Becker et al. 2023; Vogt et al. 2023; Straumann et al. 2023; Klaiber et al. 2024). So far, most analyses have focused on quantitative aspects because they enable objective, unbiased, directly comparable, and economic evaluations, which oftentimes justifies their superior scientific reputation over qualitative methods. However, the reduction of bias comes at the price of subjective information as the individual experience is reduced to a predefined theoretical construct. Given the highly personal nature of psychedelic substances and their ability to induce an experience that is tailored to the consumer’s individual biography, the evaluation of qualitative data in psychedelic research is indispensable. Consequently, qualitative research methods complement quantitative approaches and contribute to a broader understanding of clinical realities (Malterud 2001).

Qualitative data assessment in psychedelic research is usually conducted through (semi-)structured interviews. Several studies reported results of such interviews with patients who suffered from various conditions that were being treated with psychedelic substances, such as treatment-resistant depression (Watts et al. 2017), substance use disorders (Nielson et al. 2018; Noorani et al. 2018; Talin and Sanabria 2017; Loizaga-Velder and Verres 2014), distress in the context of life-threatening diseases (Belser et al. 2017; Swift et al. 2017; Gasser et al. 2015), and eating disorders (Lafrance et al. 2017; Renelli et al. 2020).

Some qualitative and quantitative research has been performed on motives and subjective experiences of recreational psychedelic drug users. The results from these studies indicate that the predominant reasons people use psychedelic substances recreationally are the desire to expand awareness, to use these experiences as coping mechanisms, and for enhancement purposes (i.e., making an existing state more fun or interesting) as reported in a recent systematic review (Basedow and Kuitunen-Paul 2022). Most studies suggest that while hedonistic motives are indeed prevalent, they are not the driving forces of recreational psychedelic use (Prepeliczay 2002; Móró et al. 2011; Soares et al. 2023; Kettner et al. 2019a). Preferred settings included familiar settings (e.g., one’s home), settings in nature, and private or public music events (Prepeliczay 2002; Pestana et al. 2021). Recreational users reported carefully choosing their “trip” companions based on the criteria of trust, familiarity, and reliability (Prepeliczay 2002). The majority of people appear to prefer the presence of a close friend, a lover, or a small group to being alone during the experience (Prepeliczay 2002; Palmer and Maynard 2022).

Taken together, several studies have outlined the motives and subjective experiences of recreational psychedelic substance users and patients who undergo psychedelic-assisted therapy through qualitative data analysis. However, motives and retrospective appraisal of healthy volunteers who participate in clinical trials investigating psychedelics remain largely unclear. Therefore, the present analysis addressed questions about why healthy people sign up for psychedelic trials, factors that contribute to an either positive or negative psychedelic experience, and circumstances under which they would want to use these substances again.

## Methods

### Study design

The present study used data from six randomized, double-blind, placebo-controlled crossover trials that were conducted in our laboratory at University Hospital Basel between 2018 and 2023. Table 1 lists the key characteristics of each trial. In all six studies, the order of substance administration was random and counterbalanced. Washout periods between sessions were at least 7 days for all studies. All studies were conducted in accordance with the Declaration of Helsinki and International Conference on Harmonization Guidelines in Good Clinical Practice and were approved by the Ethics Committee of Northwest Switzerland (EKNZ) and Swiss Federal Office for Public Health.

**Table 1:** List of studies and study characteristics

Study	Abbreviation	NCT identifier	Design	Number of participants	Number of psychedelic sessions	Duration of sessions
Comparison of LSD and psilocybin	LPS	NCT03604744	randomized	*n* = 28	*n* = 4	25 h
			double-blind			
			placebo-controlled			
			cross-over			
Comparison of LSD, psilocybin, and mescaline	LPM	NCT04227756	randomized	*n* = 32	*n* = 3	25 h
			double-blind			
			placebo-controlled			
			cross-over			
Dimethyltryptamine bolus / maintenance infusion	DMT	NCT04353024	randomized	*n* = 27	*n* = 4	4 h
			double-blind			
			placebo-controlled			
			cross-over			
Comparison of LSD, MDMA, and LSD + MDMA	LMS	NCT04516902	randomized	*n* = 24	*n* = 2	13 h
			double-blind			
			placebo-controlled			
			cross-over			
Mescaline Dose–Response	MDR	NCT04849013	randomized	*n* = 16	*n* = 5	31 h
			double-blind			
			placebo-controlled			
			cross-over			
Comparison of LSD and LSD + Ketanserin	L-Ket	NCT04558294	randomized	*n* = 24	*n* = 2	13 h
			double-blind			
			placebo-controlled			
			cross-over			

### Participants

The present study sample consisted of a total of 151 healthy volunteers (74 men and 77 women; mean age ± SD: 32 ± 5 years; range: 25–64 years). Participants were recruited through word of mouth and advertisements on the website of the University Hospital Basel. Out of the six trials, three additionally recruited participants through an advertisement on the virtual marketplace platform of the University of Basel. Each recruiting advertisement contained the same amount of information, with the content only adapted to reflect the specifics of each trial. An example of a recruiting advertisement is shown in Supplementary Image S1.

All participants were screened for pathological psychological and somatic conditions before study inclusion. Only healthy people were included. The predominant nationalities were Swiss and German. Of the 151 participants, 57 were psychedelic-naïve before study participation (i.e., they had never had a serotonergic or non-serotonergic psychedelic experience). All of the 151 volunteers who were included in the analysis completed all sessions of the study they participated in. All participants provided written informed consent and received payment for their participation.

### Study drugs

The study drugs were classic (serotonergic) psychedelics: lysergic acid diethylamide (LSD) base (0.1 and 0.2 mg), LSD base (0.1 mg) + 3,4-methylenedioxymethamphetamine (MDMA) hydrochloride (100 mg), psilocybin dihydrate (15, 20, and 30 mg), mescaline hydrochloride (100, 200, 300, 400, 500, and 800 mg), and *N*,*N*-dimethyltryptamine (DMT) hemifumarate (0.6 mg/min infusion with and without a 15 mg bolus, 1.0 mg/min infusion with and without a 25 mg bolus). MDMA alone was not considered a psychedelic substance in the present study.

### Setting

Study sessions were conducted on the first floor of a building at University Hospital Basel. The hospital room included an adjustable bed, a side table, a desk, two chairs, a sink, a trolley with medical material, and a centrifuge (see Supplementary Image S2). Participants had the option to request centrifugation of blood samples to be done in a separate room. The room had direct access to a large balcony with a view of the hospital park including various trees and plants, lawns, and a pond (see Supplementary Images S3 and S4). The participants had the opportunity to sit in a deckchair on the balcony for an unlimited amount of time or to go for an accompanied walk in the hospital park after the most intense drug effects had passed (except for the DMT study, which required participants to lie in bed for the entire 4-h session). The participants spent their sessions in bed for the most part, listening to music through headphones. Most of them turned their attention inward and closed their eyes during the most intense phase of the experience. Eyeshades were available but not mandatory, and most participants decided against them. Participants were mainly under 1:1 supervision for the entirety of the acute experience, or under 1:2 supervision in the case of trainee instruction. The DMT study (Table 1) was the only one in which two investigators were present during all of the experience due to technical requirements. The main investigator was a PhD student with a degree in psychology or pharmacy, or a medical doctor. In the studies that required the participants to spend the night after the experience in the hospital facility because of extended blood sampling, a shift change that introduced a trainee took place in the evening. The participants spent the night with the trainee investigator in the room next to theirs.

### Study procedures

The present study explored three general topics: (1) motivation for psychedelic trial participation (Research Topic 1), (2) retrospective appraisal (i.e., factors that promote/compromise a positive psychedelic experience (Research Topic 2)), and (3) conclusions of participants after study completion (Research Topic 3). The data were collected from three different sources: data for Research Topic 1 was obtained during the screening procedure that included the investigation of motivational aspects (subdivided into incentives, expectations, and hopes regarding study participation). The investigator who conducted the screening interview asked three standardized questions: a.) “Why did you sign up for this study?” (*Incentives*), b.) “What do you expect regarding your psychedelic study experience?” (*Expectations*), and c.) “Do you have any specific hopes regarding your study experience?” (*Hopes*). The LPS study (Table 1) was the only trial to cover only the *Incentives* question*.* Data for Research Topics 2 and 3 were obtained at study completion during the end of study visit when the participants completed a questionnaire with standardized, open questions. The questions for Research Topic 2 were a). “What impact did the investigator have on your experience?”, b.) “What impact did the hospital room have on your experience?”, c.) “What were you satisfied with?”, and d.) “What is in need of improvement?”. The questions for Research Topic 3 were a.) “Were your expectations regarding your study participation met, disappointed, or exceeded?”, b.) “Would you take the study substances again?”, and c.) “If yes, which setting and company would you choose?”. See Table 2 for an overview of the questions asked.

**Table 2:** Overview of the standardized, open questions for each research topic

Research Topic	Questions used	Point in time	Type of interview	Studies used
1. Motivation for psychedelic trial participation	1. "Why did you sign up for this study?"	Screening visit	Oral	LPS (first question only^a^), LPM, DMT, LMS, MDR, L-Ket
	2. "What do you expect regarding your psychedelic study experience?"			
	3. "Do you have any specific hopes regarding your study experience?"			
2. Factors promoting/compromising a positive psychedelic experience	1. "What impact did the study investigator have on your experience?"	End of study visit	Pen and paper	LPS, LPM, DMT, LMS, MDR, L-Ket
	2. "What impact did the setting have on your experience?"			
	3. "What were you satisfied with?"			
	4. "What is in need of improvement?"			
3. Conclusion of participants after study completion	1. "Were your expectations regarding your study experience met, disappointed, or exceeded?"	End of study visit	Pen and paper	LPS, LPM, DMT, LMS, MDR, L-Ket
	2. "Would you take the study substances again?"			
	3. "If yes, what setting and what company would you choose?"			

^a^In the LPS study, only the first question ("Why did you sign up for this study?") was used to determine the motivation for study participation

### Data analysis

For the data analysis, inductive category development according to Mayring’s qualitative content analysis (Mayring and Fenzl 2019) was used. To avoid potential biases, the analysis was performed using a data-driven bottom-up approach rather than a top-down approach which is based on antecedent theory building. The data were worked through several times to extract subcategories and their overarching dimensions and were analyzed according to the following order: (1) disassembly of meaning, (2) establishing analysis guidelines (see Supplementary Table S1 for details), (3) coding, (4) clustering into subcategories, (5) clustering into dimensions, (6) division into statement count and subject count, (7) division into naïve and experienced participants, and (8) final counting of each category. The data were coded with MAXQDA 22.6.1 software. Initial coding was performed by one person only. Because the data were not obtained through oral interviews and thus neither required transcription nor extensive paraphrasing, parallel coding through an intercoder was omitted. However, subcategory/dimension designation and allocation were revised by a second evaluator. Divergencies in category formation led to a joint redefinition and readjustments of subcategory/dimension constructs and content. For further information on category evolution, see Supplementary Information.

## Results

### Motivation for psychedelic trial participation (Research Topic 1)

The motivation section resulted in a total of 595 coded elements in the full sample that included both psychedelic-experienced and psychedelic-naïve participants (*n* = 151). See Table 3 for an overview of the motivation dimensions. See Supplementary Table S2 for a complete overview of the dimensions and their underlying subcategories.

**Table 3:** Motivation (Research Topic 1): List of dimensions

All participants				Experienced participants				Naïve participants			
	*Statement count*	*participant count*	*% participants*		*Statement count*	*participant count*	*% participants*		*Statement count*	*participant count*	*% participants*
*Incentives* (*n* = 151)				*Incentives* (*n* = 94)				*Incentives* (*n* = 57)			
1. Interest/fascination/curiosity for psychedelics	131	123	81.46%	1. Interest/fascination/curiosity for psychedelics	85	77	81.91%	1. Interest/fascination/curiosity for psychedelics	46	46	80.70%
2. Appealing setting	63	58	38.41%	2. Affinity to research	40	39	41.49%	2. Appealing setting	30	26	45.61%
3. Affinity to research	57	56	37.09%	3. Appealing setting	33	32	34.04%	3. Affinity to research	17	17	29.82%
4. (Self-)Exploration	36	32	21.19%	4. (Self-)Exploration	21	18	19.15%	4. (Self-)Exploration	15	14	24.56%
5. Instrumental gain	12	12	7.95%	5. Idealistic motives	7	6	6.38%	5. Instrumental gain	7	7	12.28%
6. Idealistic motives	7	6	3.97%	6. Instrumental gain	5	5	5.32%				
*Expectations* (*n* = 126)				*Expectations* (*n* = 83)				*Expectations* (*n* = 43)			
1. No expectations	45	45	35.71%	1. No expectations	30	30	36.14%	1. No expectations	15	15	34.88%
2. Personal evolvement and sensemaking	31	27	21.43%	2. Personal evolvement and sensemaking	15	14	16.87%	2. Personal evolvement and sensemaking	16	13	30.23%
3. Specific phenomenological features	20	19	15.08%	3. Positive experience	14	14	16.87%	3. Specific phenomenological features	10	9	20.93%
4. Positive experience	18	18	14.29%	4. Anticipated challenges	12	12	14.46%	4. Broadening of horizons	5	5	11.63%
5. Broadening of horizons	16	16	12.70%	5. Broadening of horizons	11	11	13.25%	5. Positive experience	4	4	9.30%
6. Anticipated challenges	13	13	10.32%	6. Specific phenomenological features	10	10	12.05%	6. Anticipated challenges	1	1	2.33%
*Hopes* (*n* = 126)				*Hopes* (*n* = 83)				*Hopes* (*n* = 43)			
1. No hopes	44	44	34.92%	1. Transformative processes	34	28	33.73%	1. No hopes	17	17	39.53%
2. Transformative processes	44	36	28.57%	2. No hopes	27	27	32.53%	2. Broadening of horizons	9	9	20.93%
3. Broadening of horizons	21	21	16.67%	3. Broadening of horizons	12	12	14.46%	3. Transformative processes	10	8	18.60%
4. Entertainment/hedonism/sensation seeking	12	10	7.94%	4. Entertainment/hedonism/sensation seeking	9	7	8.43%	4. Positive experience (nonspecific)	6	6	13.95%
5. Transcendent experience	10	9	7.14%	5. Transcendent experience	6	5	6.02%	5. Transcendent experience	4	4	9.30%
6. Positive experience (nonspecific)	10	9	7.14%	6. Positive experience (nonspecific)	4	3	3.61%	6. Entertainment/hedonism/sensation seeking	3	3	6.98%
7. Avoidance-oriented hopes	4	4	3.17%	7. Avoidance-oriented hopes	1	1	1.20%	7. Avoidance-oriented hopes	3	3	6.98%
8. In-depth look into clinical research	1	1	0.79%	8. In-depth look into clinical research	1	1	1.2%				
N/A	3			N/A	0			N/A	3		
Uncodeable	9			Uncodeable	5			Uncodeable	4		

Questions used were: 1. *Incentives*; "Why did you sign up for this study?", 2. *Expectations*; "What do you expect regarding your psychedelic experiences?", 3. *Hopes*; "Do you have any specific hopes regarding your psychedelic experiences?". For *incentives*, the data of six studies were used. For *expectations* and *hopes*, the data of five studies were used (all except LPS study). As one participant's statements may be assigned to several subcategories and several subcategories are clustered into the same dimension, the statement count does not equal the participant count. N/A; not available; missing values, Uncodeable; statements that were omitted due to content ambiguity

For *Incentives*, 31 subcategories were clustered into six dimensions (*n* = 151). For *Expectations*, 30 subcategories were clustered into six dimensions (*n* = 126). For *Hopes*, 42 subcategories were clustered into eight dimensions (*n* = 126).

When asked about *Incentives*, 81.5% of the participants in the full sample responded they had signed up for the study because of their interest, fascination, and curiosity for psychedelic substances, 38.4% thought the setting was appealing (e.g., “safe setting,” “purity and controlled content of study drug,” “opportunity to integrate the experience professionally,” “avoid illegality”), 37.1% mentioned an affinity for research (e.g., “interest in research,” “support psychedelic research”), 21.19% mentioned (self-)exploration motives (e.g., “exploring another form of consciousness,” “psychological interest,” “affinity for mindfulness/meditation”), and 8.0% referred to an instrumental gain they could draw from study participation (“financial compensation” and “writing an article to publish”). None of the participants mentioned financial compensation as the sole incentive for study participation. A total of 4.0% referred to idealistic motives (e.g., “contribute to decriminalization and destigmatization of psychedelics,” “support psychedelic renaissance”). Dividing the full sample into a subset of psychedelic-experienced participants and a subset of psychedelic-naïve participants resulted in the following percentages (Table 3): (1) interest/fascination/curiosity for psychedelic substances (81.9% of experienced participants *vs*. 80.7% of naïve participants), (2) affinity for research (41.5% of experienced participants *vs*. 29.8% of naïve participants), (3) appealing setting (34.0% of experienced participants *vs*. 45.6% of naïve participants), (4) (self-)exploration (19.2% of experienced participants *vs*. 24.6% of naïve participants), (5) idealistic motives (6.4% of experienced participants *vs*. 0% of naïve participants), and (6) instrumental gain (5.3% of experienced participants *vs*. 12.3% of naïve participants).

When asked about *Expectations*, 35.7% of the participants in the full sample responded they had no expectations about their study participation, 21.4% expected to experience some sort of personal evolvement and/or sensemaking process (e.g., “gaining new insights/perspectives,” “gaining access to the subconscious,” “resonating experience,” “informative experience”), 15.1% expected the occurrence of specific phenomenological features (e.g., “altered sensory or emotional perception,” “ego dissolution and disembodiment”), 14.3% expected their experience to be positive (e.g., “enjoyable experience,” “funny experience,” “similarity to previous positive psychedelic experiences”), 12.7% expected their horizons to broaden (e.g., “experience the unknown,” “novel experience,” “altered cognitive patterns”), and 10.3% anticipated some sort of challenges to lie ahead (e.g., “intense experience,” “physical side-effects”). Dividing the full sample into a subset of psychedelic-experienced participants and a subset of psychedelic-naïve participants resulted in the following percentages (Table 3): (1) no expectations (36.1% of experienced participants *vs*. 34.9% of naïve participants), (2) personal evolvement and sensemaking (16.9% of experienced participants *vs*. 30.2% of naïve participants), (3) positive experience (16.9% of experienced participants *vs*. 9.3% of naïve participants), (4) anticipated challenges (14.5% of experienced participants *vs*. 2.3% of naïve participants), (5) broadening of horizons (13.2% of experienced participants *vs*. 11.6% of naïve participants), and (6) specific phenomenological features (12.1% of experienced participants *vs*. 20.9% of naïve participants).

When asked about *Hopes*, 34.9% of the participants in the full sample said they had no hopes about their study participation, 28.6% hoped they would face transformative processes of some sort (e.g., “new insights/perspectives,” “spiritual experience,” “finding answers to personal issues,” “discovering new aspects of the psyche,” “self-acceptance,” “finding oneself as an individual,” “connectedness”), 16.7% hoped to be able to broaden their horizons (e.g., “better grasp of altered states of consciousness”), 7.9% hoped their experience would serve as entertainment or satisfy hedonistic/sensation-seeking purposes (e.g., “satisfy curiosity,” “finding out if the experience pleases me,” “exciting experience,” “intense substance effects,” “aesthetic experience,” “nice mental images”), 7.1% hoped for a transcendent experience (e.g., “expanding consciousness,” “escaping linear reality,” “exploring mental borders,” “recognizing what is usually inaccessible”), 7.1% hoped for a positive experience with no further specification, 3.2% referred to avoidance-oriented hopes with regard to their study participation (i.e., “no bad trip,” “not having to suffer,” “little side-effects”), and 0.8% hoped to gain an in-depth look into clinical research through their participation. Dividing the full sample into a subset of psychedelic-experienced participants and a subset of psychedelic-naïve participants resulted in the following percentages (Table 3): (1) transformative processes (33.7% of experienced participants *vs*. 18.6% of naïve participants), (2) no hopes (32.5% of experienced participants *vs*. 39.5% of naïve participants), (3) broadening of horizons (14.5% of experienced participants *vs*. 20.9% of naïve participants), (4) entertainment/hedonism/sensation seeking (8.4% of experienced participants *vs*. 7.0% of naïve participants), (5) transcendent experience (6.0% of experienced participants *vs*. 9.3% of naïve participants), (6) positive experience (nonspecific; 3.6% of experienced participants *vs*. 14.0% of naïve participants), and (7) avoidance-oriented hopes (1.2% of experienced participants *vs*. 7.0% of naïve participants).

### Retrospective appraisal: factors promoting/compromising a positive psychedelic experience (Research Topic 2)

The appraisal section resulted in a total of 597 coded elements in the full sample (*n* = 150). See Table 4 for an overview of the dimensions. See Supplementary Table S3 for a complete overview of the dimensions and their underlying subcategories. For *Positive setting aspects, 2*1 subcategories were clustered into seven dimensions. For *Negative setting aspects,* 23 subcategories were clustered into six dimensions. For *Positive investigator aspects*, 19 subcategories were clustered into five dimensions. For *Negative investigator aspects,* 27 subcategories were clustered into five dimensions. The setting factors identified to promote a positive psychedelic experience were (1) music (52.7% in the full sample), (2) access to nature (40.7%; e.g., “sitting outside with a view of the hospital park”, “walk in the park”), (3) specific room features (22%; e.g., appreciation of “blank surfaces serving as a visual projection board,” “comfort of bed,” “adjustable light conditions”, “wooden/marble wall”), (4) hospital setting (10%; e.g., “hospital setting was assuring”, “hospital setting enhanced introspection”), (5) food (8.7%, e.g., “appreciation for food”, “intensified perception of food”), (6) pleasant weather conditions (8%), and (7) appreciation for specific instruments and measurements related to conduct of the study (2.7%; e.g., certain questionnaires). Dividing the full sample into a subset of psychedelic-experienced participants and a subset of psychedelic-naïve participants resulted in the following percentages (Table 4): (1) music (53.6% of experienced participants *vs*. 50.9% of naïve participants), (2) access to nature (40.2% of experienced participants *vs*. 41.5% of naïve participants), (3) specific room features (22.7% of experienced participants *vs*. 20.8% of naïve participants), (4) hospital setting (9.3% of experienced participants *vs*. 11.3% of naïve participants), (5) pleasant weather conditions (8.3% of experienced participants *vs*. 11.3% of naïve participants), (6) food (7.2% of experienced participants *vs*. 11.3% of naïve participants), and (7) instruments and measurements (3.1% of experienced participants *vs*. 1.9% of naïve participants).

**Table 4:** Retrospective appraisal: Factors promoting/compromising a positive psychedelic experience (Research Topic 2): List of dimensions

All participants (*n* = 150)				Experienced participants (*n* = 97)				Naïve participants (*n* = 53)			
	*Statement count*	*Participant count*	*% participants*		*Statement count*	*Participant count*	*% participants*		*Statement count*	*Participant count*	*% participants*
*Positive setting aspects*				*Positive setting aspects*				*Positive setting aspects*			
1. Music	79	79	52.67%	1. Music	52	52	53.61%	1. Music	27	27	50.94%
2. Access to nature	70	61	40.67%	2. Access to nature	46	39	40.21%	2. Access to nature	24	22	41.51%
3. Specific room features	37	33	22.00%	3. Specific room features	25	22	22.68%	3. Specific room features	12	11	20.75%
4. Hospital setting	15	15	10.00%	4. Hospital setting	9	9	9.28%	4. Hospital setting	6	6	11.32%
5. Food	13	13	8.67%	5. Pleasant weather conditions	8	8	8.25%	5. Food	6	6	11.32%
6. Pleasant weather conditions	12	12	8.00%	6. Food	7	7	7.22%	6. Pleasant weather conditions	4	4	7.55%
7. Instruments and measurements	4	4	2.67%	7. Instruments and measurements	3	3	3.09%	7. Instruments and measurements	1	1	1.89%
*Negative setting aspects*				*Negative setting aspects*				*Negative setting aspects*			
1. Hospital setting	42	34	22.67%	1. Hospital setting	29	23	23.71%	1. Study requirements and measurements	12	12	22.64%
2. Study requirements and measurements	37	33	22.00%	2. Study requirements and measurements	25	21	21.65%	2. Hospital setting	13	11	20.75%
3. Restriction of movement	20	20	13.33%	3. Restriction of movement	18	18	18.56%	3. Irritating noises	5	5	9.43%
4. Specific room features	19	14	9.33%	4. Specific room features	15	11	11.34%	4. Unpleasant weather conditions	4	4	7.55%
5. Irritating noises	12	12	8.00%	5. Irritating noises	7	7	7.22%	5. Specific room features	4	3	5.66%
6. Unpleasant weather conditions	8	8	5.33%	6. Unpleasant weather conditions	4	4	4.12%	6. Restriction of movement	2	2	3.77%
*Positive investigator aspects*				*Positive investigator aspects*				*Positive investigator aspects*			
1. Trusting/secure relationship	109	103	68.67%	1. Trusting/secure relationship	74	70	72.16%	1. Trusting/secure relationship	35	33	62.26%
2. Good care taken	28	28	18.67%	2. Good care taken	17	17	17.53%	2. Good care taken	11	11	20.75%
3. Non-intruding/non-invasive behavior	25	25	16.67%	3. Non-intruding/non-invasive behavior	14	14	14.43%	3. Non-intruding/non-invasive behavior	11	11	20.75%
4. Professionality and competence	19	19	12.67%	4. Professionality and competence	10	10	10.31%	4. Professionality and competence	9	9	16.98%
5. Pleasant atmosphere	6	6	4.00%	5. Pleasant atmosphere	4	4	4.12%	5. Pleasant atmosphere	2	2	3.77%
*Negative investigator aspects*				*Negative investigator aspects*				*Negative investigator aspects*			
1. Lack of support	16	16	10.67%	1. Lack of support	9	9	9.28%	1. Lack of support	7	7	13.21%
2. Investigator induced psychological discomfort	15	15	10.00%	2. Investigator induced psychological discomfort	11	11	11.34%	2. Investigator induced psychological discomfort	4	4	7.55%
3. Contact with trainees	6	6	4.00%	3. Contact with trainees	4	4	4.12%	3. Contact with trainees	2	2	3.77%
4. Excess of care and investigator presence	4	4	2.67%	4. Excess of care and investigator presence	3	3	3.09%	4. Excess of care and investigator presence	1	1	1.89%
5. Lack of trust	1	1	0.67%	5. Lack of trust	1	1	1.03%				
N/A	1			N/A	1			N/A	0		
Uncodeable	8			Uncodeable	1			Uncodeable	7		

Questions used were: 1. "What impact did the setting have on your experience?", 2. "What impact did the investigator have on your experience?", 3. "What were you satisfied with?", 4. "What is in need of improvement?". As one participant's statements may be assigned to several subcategories and several subcategories are clustered into the same dimension, the statement count does not equal the participant count. N/A; not available; missing values, Uncodeable; statements that were omitted due to content ambiguity

The setting factors identified to compromise a positive psychedelic experience were (1) hospital setting (22.7% in the full sample; e.g., “setting was sterile,” “room felt confining”), (2) study requirements and measurements (22%; e.g., “questionnaires pulled me out of the experience,” “magnetic resonance imaging (MRI) was unpleasant,” “study sessions were too long”), (3) restriction of movement (13.3%; e.g., “restriction of movement through IV line and blood pressure cuff,” “general activity restrictions”), (4) specific room features (9.3%; e.g., “lack of room plants,” annoying sight of clock,” “uncomfortable furniture”), (5) irritating noises (8%; e.g., “centrifuge,” “clicking keyboard,” “construction site next to the hospital”), and (6) unpleasant weather conditions (5.3%). Dividing the full sample into a subset of psychedelic-experienced participants and a subset of psychedelic-naïve participants resulted in the following percentages (Table 4): (1) hospital setting (23.7% of experienced participants *vs*. 20.8% of naïve participants), (2) study requirements and measurements (21.7% of experienced participants *vs*. 22.6% of naïve participants), (3) restriction of movement (18.6% of experienced participants *vs*. 3.8% of naïve participants), (4) specific room features (11.3% of experienced participants *vs*. 5.7% of naïve participants), (5) irritating noises (7.2% of experienced participants *vs*. 9.4% of naïve participants), and (6) unpleasant weather conditions (4.1% of experienced participants *vs*. 7.6% of naïve participants).

Concerning the investigators’ behavior, the factors identified to promote a positive psychedelic experience were (1) trusting/secure relationship between participant and investigator (68.7% in the full sample; e.g., “investigator induced a sense of security,” “investigator was benevolent/caring/supportive/empathetic,” “familiar investigator,” “ I felt at ease, was able to let go”), (2) good care taken (18.7%; e.g., “investigator was responsive to participant wishes and requests”), (3) non-intruding/non-invasive behavior (16.7%; e.g., “investigator was unobtrusive,” “investigator was unbiased,” “investigator enabled autonomy”), (4) investigator being professional and competent (20.7%; e.g., “competent and reliable care,” “I was informed thoroughly,” “constantly available and experienced investigator”), and (5) investigator inducing a pleasant atmosphere (4%; e.g., “investigator induced quiet/positive atmosphere,” “investigator induced personal atmosphere”). Dividing the full sample into a subset of psychedelic-experienced participants and a subset of psychedelic-naïve participants resulted in the following percentages (Table 4): (1) trusting/secure relationship (72.2% of experienced participants *vs*. 62.3% of naïve participants), (2) good care taken (17.5% of experienced participants *vs*. 20.8% of naïve participants), (3) non-intrusive/non-invasive behavior (14.4% of experienced participants *vs*. 20.8% of naïve participants), (4) professionality and competence (10.3% of experienced participants *vs*. 17.0% of naïve participants), and (5) pleasant atmosphere (4.1% of experienced participants *vs*. 3.8% of naïve participants).

Concerning the investigators’ behavior, the factors identified to compromise a positive psychedelic experience were (1) lack of support (10.7% in the full sample; e.g., “I was not given enough guidance/reassurance,” “I was not given enough food,” “investigator did not check in enough,” “experience was not integrated properly,” and “investigator was turned away physically”), (2) investigator induced psychological discomfort (10%; e.g., “I felt watched,” “physiological measurements were not announced,” “I felt treated like a child,” “I would have preferred an older investigator with more life experience,” “I would have preferred one man and one woman as investigators,” “I wasn’t informed properly about the dosage intensity,” “respiratory face mask of investigator,” “investigator was not calm enough in difficult situation,” “investigator talked about negative things at the beginning of the session,” “investigator was hectic,” and “investigator studied questionnaires too closely, induced paranoia”), (3) contact with trainees (4%; e.g., “shift change, arrival of trainee investigator,” “night shift was psychedelic-naïve,” and “trainees were insecure”), (4) excess care and investigator presence (2.7%; e.g., “I would have preferred more time alone,” “atmosphere induced by investigator was too personal,” and “investigator presence was bothering”), and (5) lack of trust (0.7%). Dividing the full sample into a subset of psychedelic-experienced participants and a subset of psychedelic-naïve participants resulted in the following percentages (Table 4): (1) lack of support (9.3% of experienced participants *vs*. 13.2% of naïve participants), (2) investigator induced psychological discomfort (11.3% of experienced participants *vs*. 7.6% of naïve participants), (3) contact with trainees (4.1% of experienced participants *vs*. 3.8% of naïve participants), (4) excess care and investigator presence (3.1% of experienced participants *vs*. 1.9% of naïve participants), and (5) lack of trust (1.0% of experienced participants *vs*. 0% of naïve participants).

### Conclusions of participants after study completion (Research Topic 3)

The conclusions section resulted in a total of 604 coded elements in the full sample (*n* = 149). In this section, statement count and subject count were equivalent throughout. The first two questions (“Were expectations met, disappointed, or exceeded?” and “Would you take the study substances again?”) resulted in categorical coding (i.e., each participant’s statement can only be found in a single, independent subcategory, whereas in the two following questions (“What setting would you choose for a future psychedelic experience?” and “What company would you choose for a future psychedelic experience?”), a participant’s response may have been assigned to several subcategories at once. See Table 5 for an overview of the dimensions. See Supplementary Table S4 for a complete overview of the dimensions and their underlying subcategories.

**Table 5:** Conclusions (Research Topic 3): List of dimensions

All participants (*n* = 149)				Experienced participants (*n* = 96)				Naïve participants (*n* = 53)			
	*Statement count*	*participant count*	*% participants*		*Statement count*	*participant count*	*% participants*		*Statement count*	*participant count*	*% participants*
*Were expectations met, disappointed, or exceeded?*				*Were expectations met, disappointed, or exceeded?*				*Were expectations met, disappointed, or exceeded?*			
Exceeded	66	66	44.30%	Exceeded	38	38	39.58%	Exceeded	28	28	52.83%
Met	42	42	28.19%	Met	31	31	32.29%	Met	11	11	20.75%
Mixed, depending on study day	15	15	10.07%	Disappointed	9	9	9.38%	Mixed, depending on study day	7	7	13.21%
Disappointed	13	13	8.72%	Mixed, depending on study day	8	8	8.33%	Disappointed	4	4	7.55%
N/A but overall positive experience	7	7	4.70%	N/A but overall positive experience	5	5	5.21%	N/A but overall positive experience	2	2	3.77%
Neutral/I did not have any expectations	6	6	4.03%	Neutral/I did not have any expectations	5	5	5.21%	Neutral/I did not have any expectations	1	1	1.89%
Uncodeable	0	0	0%	Uncodeable	0	0	0%	Uncodeable	0	0	0%
N/A	0	0	0%	N/A	0	0	0%	N/A	0	0	0%
*Would you take the study substances again?*				*Would you take the study substances again?*				*Would you take the study substances again?*			
Yes	133	133	89.26%	Yes	86	86	89.58%	Yes	47	47	88.68%
Yes but not immediately	7	7	4.70%	Yes but not immediately	6	6	6.25%	Maybe	4	4	7.55%
Maybe	7	7	4.70%	Maybe	3	3	3.13%	Yes but not immediately	1	1	1.89%
No	2	2	1.34%	No	1	1	1.04%	No	1	1	1.89%
Uncodeable	0	0	0%	Uncodeable	0	0	0%	Uncodeable	0	0	0%
N/A	0	0	0%	N/A	0	0	0%	N/A	0	0	0%
*What setting would you choose?*				*What setting would you choose?*				*What setting would you choose?*			
In nature	83	83	55.70%	In nature	53	53	55.21%	In nature	30	30	56.60%
At home/familiar environment	23	23	15.44%	At home/familiar environment	15	15	15.63%	At home / familiar environment	8	8	15.09%
Clinical study setting	20	20	13.42%	Clinical study setting	12	12	12.50%	Clinical study setting	8	8	15.09%
Quiet/cozy setting (not further specified)	15	15	10.10%	Quiet/cozy setting (not further specified)	9	9	9.38%	Quiet/cozy setting (not further specified)	6	6	11.32%
N/A	10	10	6.71%	N/A	8	8	8.33%	Safe/protected setting (not further specified)	4	4	7.55%
Safe/protected setting (not further specified)	9	9	6.04%	Ceremonial setting	7	7	7.29%	Ceremonial setting	2	2	3.77%
Ceremonial setting	9	9	6.04%	Safe/protected setting (not further specified)	5	5	5.21%	Public events	2	2	3.77%
Public events	4	4	2.68%	Uncodeable	3	3	3.13%	N/A	2	2	3.77%
Uncodeable	4	4	2.68%	Public events	2	2	2.08%	Uncodeable	1	1	1.89%
Therapeutic setting	1	1	0.67%	Therapeutic setting	1	1	1.04%				
*What company would you choose?*				*What company would you choose?*				*What company would you choose?*			
With friends/familiar people	79	79	53.02%	With friends/familiar people	47	47	48.96%	With friends/familiar people	32	32	60.38%
Under observation	34	34	22.82%	Under observation	21	21	21.88%	Under observation	13	13	24.53%
N/A	20	20	13.42%	N/A	16	16	16.67%	N/A	4	4	7.55%
In a group	10	10	6.71%	In a group	8	8	8.33%	Alone	3	3	5.66%
Alone	8	8	5.37%	Alone	5	5	5.21%	In a group	2	2	3.77%
With romantic partner	6	6	4.03%	With romantic partner	4	4	4.17%	With romantic partner	2	2	3.77%
With substance-experienced company	5	5	3.36%	With substance-experienced company	4	4	4.17%	With substance-experienced company	1	1	1.89%
Uncodeable	2	2	1.34%	Uncodeable	2	2	2.08%	Uncodeable	0	0	0%

The questions "Were your expectations regarding your study experience met, disappointed, or exceeded?" and "Would you take the study substances again?" resulted in categorical coding, i.e. each participant's response was assigned to a single independent subcategory. The questions "What setting would you choose for a future psychedelic experience?" and "What company would you choose for a future psychedelic experience?" resulted in multiple category assignment, i.e., a participant's response may have been assigned to several subcategories at once. N/A; not available; missing values, Uncodeable; statements that were omitted due to content ambiguity

39.6% of the experienced participants (*n* = 96) *vs*. 52.8% of the naïve participants (*n* = 53) reported their expectations were exceeded, 32.3% of the experienced participants *vs*. 20.8% of the naïve participants felt their expectations were met, and 9.4% of the experienced participants *vs*. 7.6% of the naïve participants concluded they were disappointed. Reasons for disappointment were the following: “experience was not intense enough” (*n* = 7), “strong physical side effects” (*n* = 2), “I expected more typical psychedelic effects like insights/connectedness/visual effects” (*n* = 2), “experience was too intense” (*n* = 1), “wrong setting” (*n* = 1), “lack of euphoria” (*n* = 1), and “expectations were not met” (nonspecific; *n* = 1). 8.3% of the experienced participants *vs*. 13.2% of the naïve participants were unable to choose a clear answer because their experiences varied from session to session, 5.2% of the experienced participants *vs*. 3.8% of the naïve participants considered the experience to have been overall positive but refrained from addressing specific expectations, and 5.2% of the experienced participants *vs*. 1.9% of the naïve participants insisted that they did not have any expectations regarding their study experience.

89.6% of the experienced participants *vs*. 88.7% of the naïve participants concluded they would ingest the study substances again, 6.3% of the experienced participants *vs*. 1.9% of the naïve participants said they would ingest them again after some time had passed, 3.1% of the experienced participants *vs*. 7.6% of the naïve participants were undecided, and 1.0% of the experienced participants *vs*. 1.9% of the naïve participants replied they would not take the study substances again.

For the preferred setting for a future psychedelic experience, eight dimensions were identified: (1) in nature (55.7% in the full sample; e.g., “in nature,” “with access to nature”), (2) at home/familiar environment (15.4%), (3) clinical study setting (13.4%; e.g., “clinical study setting,” “in a legal context”), (4) quiet/cozy setting (not further specified; 10.1%), (5) safe/protected setting (not further specified; 6.0%), (6) ceremonial setting (6.0%), (7) public events (2.7%; e.g., “night life,” “music festival,” “museum”), and (8) therapeutic setting (0.7%). Dividing the full sample into a subset of psychedelic-experienced participants and a subset of psychedelic-naïve participants resulted in the following percentages: (1) in nature (55.2% of experienced participants *vs*. 56.6% of naïve participants), (2) at home/familiar environment (15.6% of experienced participants *vs*. 15.1% of naïve participants), (3) clinical study setting (12.5% of experienced participants *vs*. 15.1% of naïve participants), (4) quiet/cozy setting (not further specified; 9.4% of experienced participants *vs*. 11.3% of naïve participants), (5) ceremonial setting (7.3% of experienced participants *vs*. 3.8% of naïve participants), (6) safe/protected setting (not further specified; 5.2% of experienced participants *vs*. 7.6% of naïve participants), (7) public events (2.1% of experienced participants *vs*. 3.8% of naïve participants), and (8) therapeutic setting (1.0% of experienced participants *vs*. 0% of naïve participants).

For the preferred company for a future psychedelic experience, six dimensions were identified: (1) with friends/familiar people (53.0% in the full sample), (2) under observation (22.8%; e.g., “with study investigator,” “with sober trip sitter,” “with guide or shaman,” “with therapist”), (3) in a group (6.7%; e.g., “with happy and like-minded people,” “in a calm and reflective group of people”), (4) alone (5.4%), (5) with romantic partner (4.0%), and (6) with substance-experienced company (3.4%). Dividing the full sample into a subset of psychedelic-experienced participants and a subset of psychedelic-naïve participants resulted in the following percentages: (1) with friends/familiar people (49.0% of experienced participants *vs*. 60.4% of naïve participants), (2) under observation (21.9% of experienced participants *vs*. 24.5% of naïve participants), (3) in a group (8.3% of experienced participants *vs*. 3.8% of naïve participants), (4) alone (5.2% of experienced participants *vs*. 5.7% of naïve participants), (5) with romantic partner (4.2% of experienced participants *vs*. 3.8% of naïve participants), and (6) with substance-experienced company (4.2% of experienced participants *vs*. 1.9% of naïve participants).

## Discussion

The present analysis investigated the motivation and retrospective appraisal of healthy volunteers participating in psychedelic Phase-I trials. Unsurprisingly, around 80% of the participants in the present analysis reported having signed up for a psychedelic Phase-I trial because of their interest, fascination, and curiosity for psychedelic substances. Over 35% of all participants mentioned their affinity for research. Nearly half of psychedelic-naïve participants (45.6%) and more than one third of psychedelic-experienced participants (38.4%) were intrigued by our hospital setting because it was associated with physiological and psychological safety. While the incentive for (self-)exploration was slightly more frequent in naïve (24.6%) compared to experienced (19.8%) participants, only the experienced subset expressed idealistic aspirations (4%). Financial compensation was not a driving motivational force in either of the subsets.

Approximately one third of the participants responded they had no expectations about their study experience. More often than experienced participants (16.9%), naïve participants (30.2%) expected personal evolvement/sensemaking processes and specific phenomenological features (12.1% *vs*. 20.9%) to take place. In contrast, experienced participants more frequently expected their experience to be positive (16.9% *vs*. 9.3%) but also anticipated potential challenges more often (14.5% *vs*. 2.3%). Only one participant in the naïve subset considered the potential emergence of challenging experiences. These findings illustrate how experienced volunteers rely on their previous psychedelic experiences to form their expectations, whereas naïve volunteers are likely to cite what they have learned about psychedelics through the media or a community in which certain narratives circulate repeatedly. Such collective feedback structures (positive information derived from trials influencing the media, and the media influencing trial expectations in return) may shape trial outcomes (Noorani et al. 2023). Inflated media- or community-fueled expectations should be identified and relativized before the first study session, with a particular emphasis on potential negative acute effects.

Over one third of the participants reported not having any hopes about their study experience. Experienced participants (33.7%) more frequently than naïve participants (18.6%) hoped for transformative processes to occur, whereas more naïve participants (14.0%) than experienced participants (3.6%) hoped for a nonspecific positive experience. Entertainment/hedonism/sensation seeking was not a driving motivational force in either of the subsets (approximately 8% in the full sample). Altogether, the results indicate that the majority of volunteers participate in a psychedelic trial in search of personal development rather than hedonism or external gain.

The two predominant factors that were considered to positively influence a psychedelic experience were music (approximately 50% of the participants in both subsets) and access to nature (approximately 40% in both subsets). The use of music in psychedelic-assisted therapy was described early on, and its catalyzing effects for internal processes remain undisputed (Bonny and Pahnke 1972; Johnson et al. 2008; Kaelen et al. 2018; O’Callaghan et al. 2020). Likewise, the importance of nature has been repeatedly discussed before, with authors reporting an association between psychedelics and biophilia or nature connectedness (Kettner et al. 2019b; Newton and Moreton 2023; Irvine et al. 2023). Approximately 20% of the participants in each subset referred to specific room features that positively influenced their experience. The most frequently appreciated room feature was some sort of blank surface that served as a visual projection board.

Although the hospital setting had motivated over one third of the volunteers to participate, the “sterile hospital atmosphere” was the most frequently criticized setting aspect in hindsight (over 20% in each subset). Room features that were most commonly considered bothersome were a lack of room plants and direct sight of the clock. These findings align with the safety guidelines for psychedelic research by Johnson et al. (2008), which strongly advise against an overly sterile atmosphere to counteract hospital associations and avoid paranoia. The present analysis confirms that despite their initial beliefs, the majority of volunteers did not consider a sterile hospital room to be the ideal setting for a psychedelic experience. However, it was not further assessed which specific aspects of the psychedelic experience were considered to be compromised through the hospital setting. Overall, very few paranoid reactions were observed in our studies in this setting. Further aspects that were criticized were the data acquisition requirements of the study (over 20% in each subset). Measurements that caused the most distress were the pen and paper questionnaires that pulled the participants out of the psychedelic experience. These questionnaires that are administered as visual analog scales have proven to be one of the simplest, most accurate, and most valid instruments in the context of pharmacokinetic-pharmacodynamic studies, and are thus indispensable. However, with the present results in mind, the number of items is best reduced to a minimum. Restriction of movement through the intravenous line and blood pressure cuff was considerably more bothersome to experienced participants (18.6%) than to naïve participants (3.8%). Lastly, unpleasant weather conditions (5.3% of all participants) and uncontrollable noises (e.g., nearby construction site, centrifuge, or keyboard; 8% of all participants) were considered bothersome.

Nearly 70% of the participants (72.2% of experienced participants *vs*. 62.3% of naïve participants) mentioned the importance of the trusting/secure relationship they had with their investigator. This highlights the secure interpersonal bond as a prerequisite for an affirmative psychedelic experience not only for patients but also for healthy volunteers. The present data support the emphasis that is repeatedly placed on the participant-investigator, patient-therapist, or consumer-sitter relationship by various authors (Johnson et al. 2008; Phelps 2017; Thal et al. 2022). Nearly 20% of the participants specifically mentioned their appreciation for the good care they had received (i.e., that the investigator was responsive to participant wishes and requests), 14.4% of experienced participants and 20.8% of naïve participants explicitly expressed their appreciation for the investigator being non-intruding/non-invasive toward them, and 3.1% of experienced participants *vs*. 1.9% of naïve participants were even bothered by the constant presence of the investigator.

The most frequently criticized aspect about the investigator was a perceived lack of support in around 10% of participants, and another 10% reported the psychological discomfort that the investigator induced (e.g., the participants felt watched or the investigator’s behavior contributed to them feeling awkward or paranoid). These results illustrate the inherent balancing act that a psychedelic study investigator faces. Building and maintaining a trusting relationship and conveying a sense of security must be accompanied by sensitivity to notice when it is time to retract and give the participant space. As most participants turn inward and become reticent with the onset of subjective effects, it is not always immediately obvious when a participant feels uncomfortable. Moreover, the investigator must be aware that they may be a source of psychological discomfort for some participants as they become the vessel for projection, attributions, and sometimes misjudgment through the psychedelically altered perception of the participant. Nevertheless, it is the responsibility of the investigator to minimize situations that could potentially lead to psychological discomfort. During the sessions, investigators must be cognitively vigilant, emotionally available, and mindful in their speech, movements, gaze, and performance of study procedures. Additionally, a comprehensive information procedure that precedes the first substance session and not only covers potential psychological benefits but also stresses acute psychological challenges is particularly crucial for naïve participants. Considering that the investigator operates in the participant’s personal space and accompanies them during potentially vulnerable situations, the investigator must be able to adapt to the individual preferences and aversions (“Do’s and Don’ts”) of the participant. Individual preferences merit systematic evaluation at the screening visit. Furthermore, it is advisable to use a standardized integration approach not only for patients but also for healthy volunteers to support the processing of potentially difficult experiences and enable synchronization between perceptions of the participant and the investigator (Bathje et al. 2022).

Conclusions of the participants were mostly positive. More than a third of experienced participants and more than half of naïve participants considered their expectations exceeded, and less than 10% of each group were disappointed. Importantly, although approximately one third of the participants reported having no expectations towards their study experience at baseline, only six participants maintained this stance at the end of study visit. This finding has direct implications for psychedelic trials. Not only may expectancy effects threaten the validity of the research interpretation, but psychedelic treatment is highly susceptible to large placebo and nocebo effects that may exacerbate clinical symptoms (Szigeti and Heifets 2024; Aday et al. 2022). Similarly, expectation disappointment following inflated expectations towards treatment success may potentially aggravate symptomatology in psychiatric populations. This raises questions about the responsibility of psychedelic researchers and therapists to ensure participants are fully informed about potential outcomes. Thus, researchers and clinicians are encouraged to adopt a persistent inquiry manner to detect expectations as well as expectancy development over time, e.g., in the form of longitudinal assessments using a visual analog scale during preparatory, treatment, and integration sessions (Colloca et al. 2023). Ideally, the same investigator conducts the expectation assessment for all participants to ensure standardization.

Nearly 94% of both subsets combined said they would take the administered substances again, whereas 7 participants (4.7%) were undecided and only two participants (1.3%) said they would not. More than half of all participants would choose a setting in nature for a future psychedelic experience. The next most frequently mentioned setting options were at home or a clinical study. Ceremonial settings or public events did not attract substantial interest. More than half of all participants (53%) would prefer to use psychedelics with friends or familiar people. Approximately 20% of all participants would prefer to experience psychedelics with someone who provides supervision (e.g., study investigator, trip sitter, guide, therapist). Options that were rarely mentioned were a group setting (*n* = 10), tripping alone (*n* = 8), or tripping with a romantic partner (*n* = 6). Altogether, the results indicate that the majority of volunteers were satisfied with their study experience despite preferring different circumstances for a future psychedelic experience. It appears that research settings are used as learning fields by people with an affinity for psychedelics to prepare themselves for the private recreational use of these substances.

### Strengths and limitations

The strengths of the present study include the use of data from six standardized, high-quality trials, all of which were conducted in a randomized, double-blind, placebo-controlled, crossover design and thus the transparent traceability to documented events. The use of open questions allowed participants to respond freely and according to their own personal mode of expression. The large sample size in this study allowed separate analyses of psychedelic-experienced and psychedelic-naïve volunteers.

The present study also had several limitations. The setting was uniform with very limited ambient and social variation. The number of experienced and naïve participants was not equal. The data on participants’ motivation were assessed orally and noted by the investigator, and may be subject to alterations by the investigator’s wording. In total, seven investigators, some of whom had no formal psychological or psychiatric background, were involved in the screening procedures. Therefore, suggestive elements of inquiry may potentially have occurred. In most cases, the investigator who conducted the study sessions was the same person to conduct the end of study visit. Hence, some responses may have been influenced by social desirability. Potential differences between the psychedelic substances as well as potential differences between low, intermediate, and high doses were not analyzed. The analysis was exploratory rather than founded on a common theoretical denominator. Lastly, external validity of the present analysis may be limited because our trials entail highly specific setting conditions.

## Conclusion

The primary motivations for healthy volunteers participating in a psychedelic Phase-I trial included interest/fascination for the substances, a setting that is perceived as safe, a general affinity for research, and the opportunity for (self-)exploration. Experienced participants were more likely to hope for transformative psychological processes. The results of the present analysis indicate that most volunteers participate in a psychedelic trial in search of personal development. Money and hedonism were negligible motivational factors. Naïve participants were more likely to expect personal evolvement, sensemaking processes, and the occurrence of specific phenomenological features, which may have been fueled by media coverage. Participant expectations should be carefully assessed and relativized before the first session, and emphasis should be placed on potential psychological challenges that lie ahead. The most important setting factors that promoted a positive psychedelic experience were music and access to nature. In contrast, the most criticized setting factor was the sterility of the rooms that were used in the present studies. The most crucial positive interpersonal aspect was having a trusting/secure relationship with the investigator. The most criticized interpersonal aspects were the investigator not providing sufficient support and inducing psychological discomfort. A psychedelic investigator must master the skill of conveying a secure atmosphere without acting intrusively. Each participant’s individual preferences and aversions (“Do’s and Don’ts”) should be assessed before the first substance session. The vast majority of participants would take the study substances again, preferably with friends in nature. The present results are consistent with existing research that underscores the importance of four pivotal factors to be considered for psychedelic study experiences: (1) a secure interpersonal relationship, (2) an aesthetically pleasing environment, (3) access to nature, and (4) music.

The results of these analyses could serve as additional orientation for future psychedelic Phase-I trials to enable adequate participant centering, ensure maximal psychological safety, and enhance research standards for healthy people in psychedelic states of consciousness.

## Supplementary Information

Below is the link to the electronic supplementary material.

Supplementary file1 (DOCX 17300 KB)

## Data Availability

The datasets presented in this manuscript are not readily available because the data associated with this work are owned by the University Hospital Basel and were licensed by Mind Medicine. Requests to access the datasets should be directed to MEL (matthias.liechti@usb.ch).
