# Supplementary material for: Motivation and retrospective appraisal of psychedelic study participation: a qualitative study in healthy volunteers
Source: Psychopharmacology (Berl). 2025 Mar 26;242(8):1875–92. doi: 10.1007/s00213-025-06772-4 (PMC12296967; doi:10.1007/s00213-025-06772-4)
Supplement: Supplementary file 1 — Supplementary file1 (DOCX 17300 KB) [file 213_2025_6772_MOESM1_ESM.docx]

**Supplementary Materials**

Motivation and retrospective appraisal of psychedelic study participation: A qualitative study

in healthy volunteers

Laura Ley,^1, 2^ Matthias E. Liechti,^1, 2^ Anna M. Becker,^1, 2^ Isabelle Straumann,^1, 2^ Aaron Klaiber,^1, 2^ Friederike Holze,^1, 2^ Severin B. Vogt,^1, 2^ Denis Arikci,^1, 2^

Yasmin Schmid^1, 2^ *

^1^Clinical Pharmacology and Toxicology, Department of Biomedicine and Department of Clinical Research, University Hospital Basel; ^2^Department of Pharmaceutical Sciences, University of Basel.

*Correspondence: Yasmin Schmid, MD, Clinical Pharmacology, University Hospital Basel, Schanzenstrasse 55, Basel, CH-4031, Switzerland;

E-mail: yasmin.schmid@usb.ch; Phone: [+41 61 328 68 47](tel:+41613286866)

Submitted to *Psychopharmacology*

** Supplementary Image S1: Recruiting Advertisement of the LPM study

- 1. **Category Evolution**

Subcategory evolution is illustrated by screening interview excerpts of the motivation assessment of six LPM study participants (all screenings conducted by the same investigator). Participant designations are shown in encrypted form.

Question 1: *“Why did you sign up for this study?” (incentives)*

Question 2: *“What do you expect regarding your psychedelic study* experience*?” (expectations)*

Question 3: *“Do you have any specific hopes regarding your study experience?” (hopes)*

**LPM-00D**

Translation:

*(1) Positive experience in L-Ket study, interest in psilocybin and mescaline. Safe setting. Financial compensation*

*(2) No expectations.*

*(3) Positive experience with little side-effects.*


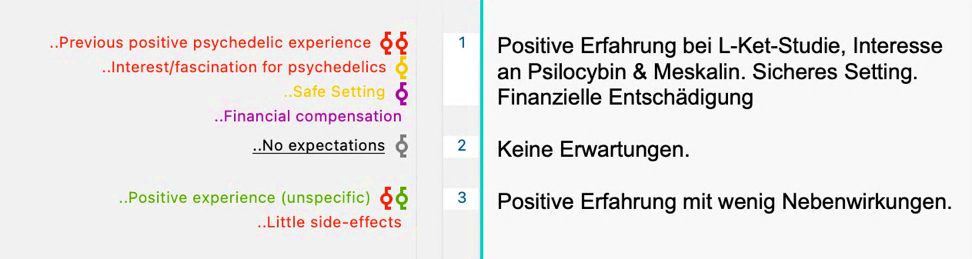


**LPM-00T**

Translation:

*(1) Curiosity regarding sensory effects, depth-psychological interest*

*(2) Hallucinations, new insights into reality/space/time. That it is going to be funny, not unpleasant.*

*(3) Transcendent experience.*


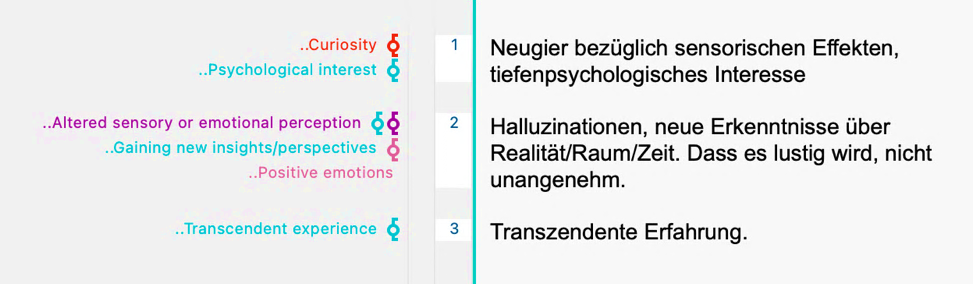


**LPM-00W**

Translation:

*(1) Interested in experiencing psychedelics firsthand – medical interest*

*(2) – pleasant, cool experience, access to the unconscious, experience something one does not know.*

*(3) No specific expectation/hope*


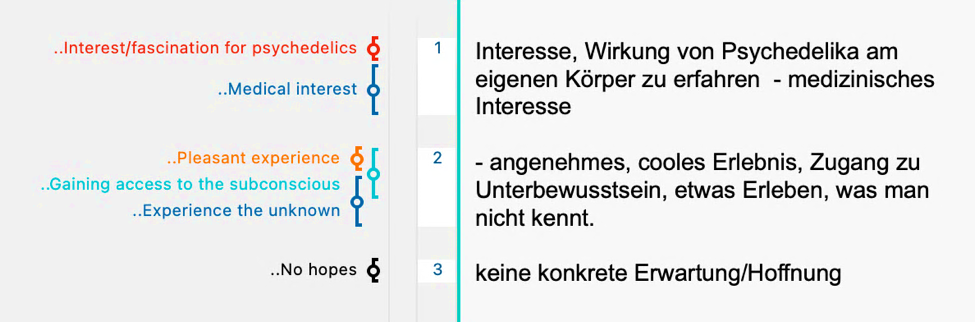


**LPM-00J**

Translation:

*(1) Curiosity for the topic, internship in […] (research group […])*

*(2) Self-interest to view certain personal insecurities from a new perspective*

*(3) Acceptance, enhanced self-esteem*


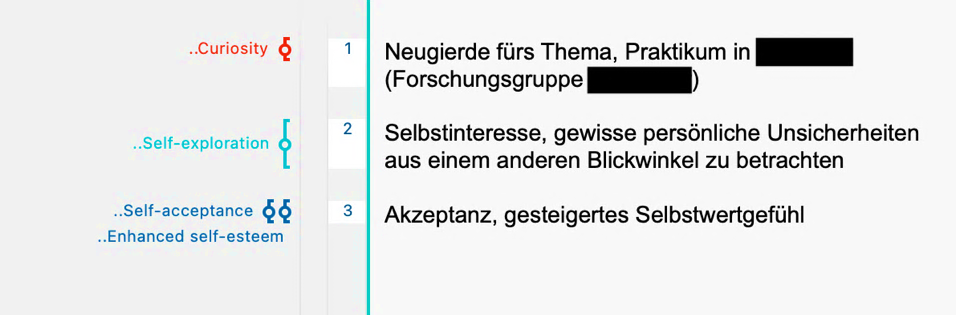


**LPM-00G**

Translation:

*(1) Interested to try these substances in a medical setting. Reliable, safe setting. Interest in research.*

*(2) “I’ll let myself be surprised.”*

*(3) No hopes*


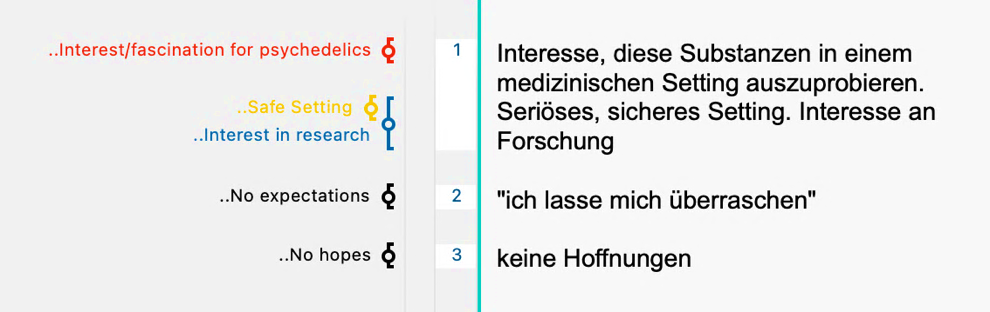


**LPM-00S**

Translation:

*(1) Michael Pollan – How to change your mind. Need for contributing to research because it is for a good cause.*

*(2) Altered perception.*

*(3) Surprising insights through altered state of consciousness. Escaping “linear reality”; novel access to art and music.*


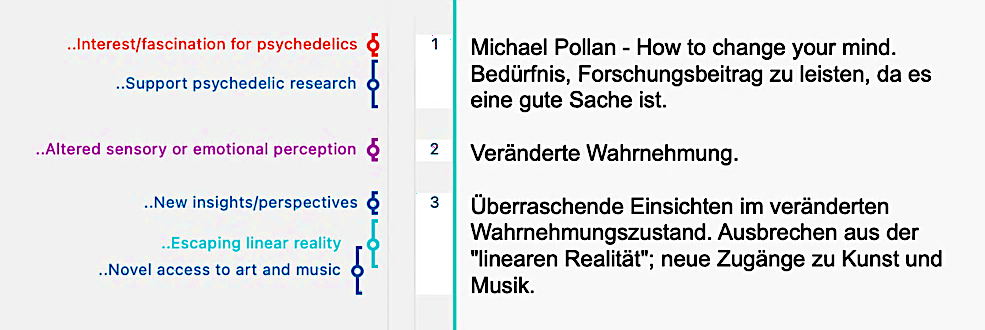


The examples above show that to form the initial subcategories, participants’ statements were a) adopted verbatim, or b) condensed as little as possible to preserve their meaning. Due to the conservative approach, parallel coding through an intercoder was omitted in this step of the procedure. However, a second evaluator was involved in the clustering of the subcategories into dimensions. First, a consensus was developed on the relatedness of single subcategories and on the identification of common denominators (illustrated by the colors of the subcategories in MAXQDA). Second, a consensus was developed on the nature of the common denominator, resulting in the designations of the overarching dimensions. Example based on the six excerpts above:

Dimension

‘Personal evolvement and sensemaking’

Subcategory clustering

‘Gaining new insights/perspectives

‘Gaining access to the subconscious’

‘Self-exploration’’

Common denominators

- Aiming for psychological expansion

- Active willingness for enhanced self-knowledge

**→  →**

**→**

**1.2 Borderline cases**

In line with a conservative interpretation approach, statements with an inextricable ambiguity were excluded from the analysis. The following examples show cases of statements that were considered uncodeable due to content ambiguity:

Question: *“What impact did the investigator have on your experience?*

Answers:

**(1) LPM-00Z**: *«Durchaus beruhigend, als Verbindung zur Realität. An Tag 1 war sie jedoch auch Teil einer «liebevollen» Verschwörung. Allenfalls etwas schwierig den Afterglow voll auszukosten. Man ist durchaus wieder in der Lage sich zu unterhalten und dies nicht zu tun kommt etwas asozial vor.»*

Translation: *“Quite calming, like a connection to reality. Though on day 1, she was also part of a “loving” conspiracy. Perhaps it was a bit difficult to enjoy the afterglow fully. One is quite capable of having a conversation again, and not doing so appears a bit antisocial.”*

Reasoning for applying label ‘uncodeable’:

The participant’s perspective on the investigator appears conflicted. On the one hand, the participant explicitly describes the investigator’s presence as calming, but on the ither hand they hint at a paranoid element (a conspiracy) that seems to have characterized the participant’s perception of the relationship. Furthermore, the statement also suggests some sort of social insecurity/inhibition/self-consciousness in that it appears that the participant would have preferred to spend more time by themselves and to avoid talking to the investigator but failed to clearly communicate this need during the sessions. The most appropriate subcategory for this statement would be ‘Investigator-induced psychological discomfort’. Yet the participant’s statements remain ambiguous, precluding a definitive categorization. Accordingly, the label “uncodeable” was assigned.

**(2) DMT-00Q**: *«Die anwesenden VersuchsleiterInnen hatten einen psychischen Einfluss auf mich, da ich ihre Schwingungen wahrnehmen konnte. Ein/e ruhige/r und fürsorgliche/r VersuchsleiterIn hatte einen positiven Einfluss auf mich.»*

Translation: *“The present investigators had a psychological impact on me as I could perceive their vibes. A calm and caring investigator had a positive influence on me.”*

Reasoning for applying label “uncodeable”:

The participant’s statement is unclear as to whether the investigator’s impact was perceived as positive or negative (the term “psychological influence” is neutral). The second sentence is ambiguously phrased due to the use of both masculine and feminine designations: “Ein/e ruhige/r und fürsorgliche/r VersuchsleiterIn” could mean: “There was one single investigator who had a positive impact on me and whose gender I am not going to reveal” or “Generally, if an investigator (regardless of gender) was calm and caring, they had a positive impact on me”. The statement carries an undertone of investigator distinction, suggesting that some investigator(s) may have been perceived as calmer and more caring than others. Due to this ambiguity, the label “uncodeable” was assigned.

Question*: “What impact did the hospital room have on your experience?”*

Answers:

**(1) LPM-00P**: *“Während Peak wenig Einfluss. Während des Ausklingens entscheidender. Kein bestimmtes Element.”*

Translation: *«Little impact during peak effects. During comedown more crucial. No specific element.»*

Reasoning for applying label “uncodeable”:

The participant’s statement emphasizes the impact of the setting but does not elaborate. The statement is, therefore, not informative enough to be interpreted. Accordingly, the label «uncodeable» was assigned.

**(2) MDR-00V**: *“Bei Uhr bin ich unschlüssig. Vielleicht sollte die nicht zentral im Blickfeld, sondern z.B. ganz links/rechts im Raum sein (oder vielleicht gar keine Uhr). Andererseits erlebt man auch viel Spannendes mit der Uhr unter Substanz.”*

Translation: *«I am not sure about the clock. Maybe it should not be placed centrally in the field of vision but, e.g., at the far right/left of the room (or maybe no clock at all). On the other hand, one experiences many interesting things with the clock under the influence of substances.»*

Reasoning for applying label “uncodeable”:

The participant’s statement identifies the clock as a crucial setting element in their experience. Based on the statement, a slight tendency towards negative associations with the clock may be inferred, yet the term “interesting” mitigates this interpretation. Because of the inconsistency, this statement was categorized as “uncodeable”.

Question: *“Would you take the study substances again? If yes, which setting and company would you choose?”*

Answers:

**(1) DMT-00U**: «Ja, in Bolus-Form mit Leuten, die vertraut sind damit. In einem angenehmen Setting.”

Translation: *«Yes, in the form of a bolus with people who are well acquainted with it. In a pleasant setting.”*

Reasoning for applying label “uncodeable”:

5

The participant’s statement clearly expresses that they would ingest the study substance(s) again (dimension *‘yes’*) and would prefer to do so with people who are experienced with the substance (dimension *‘with substance-experienced company’*). However, it is not specified how they define a “pleasant” setting. The statement therefore contains three codeable elements, two of which were further analyzed while the label “uncodeable” was assigned to the part referring to the specifics of the setting.

**(2) LMS-00N**: *«Sicher, immer wieder gerne. Optimal ist ein ruhiges Setting, wo man für sich ist, aber auch die Möglichkeit hat nach draussen in die Natur zu gehen.»*

Translation: *«Sure, anytime. Ideally in a quiet setting where you can be on your own but also have the opportunity to go outside into nature.»*

Reasoning for applying label “uncodeable”:

The participant’s statement clearly expresses that they would ingest the study substance again (dimension *‘yes’*) and would prefer to do so in a quiet setting (subcategory *‘quiet/cozy setting (not further specified)’)* with access to nature (subcategory *‘with access to nature’*). However, the statement is unclear as to whether the person would prefer to have a substance experience in a group of people or alone (the German word “man” may refer to one person or several people). Therefore, the statement contains three codeable elements, two of which were further analyzed while the label “uncodeable” was assigned to the part referring to the company.”

**
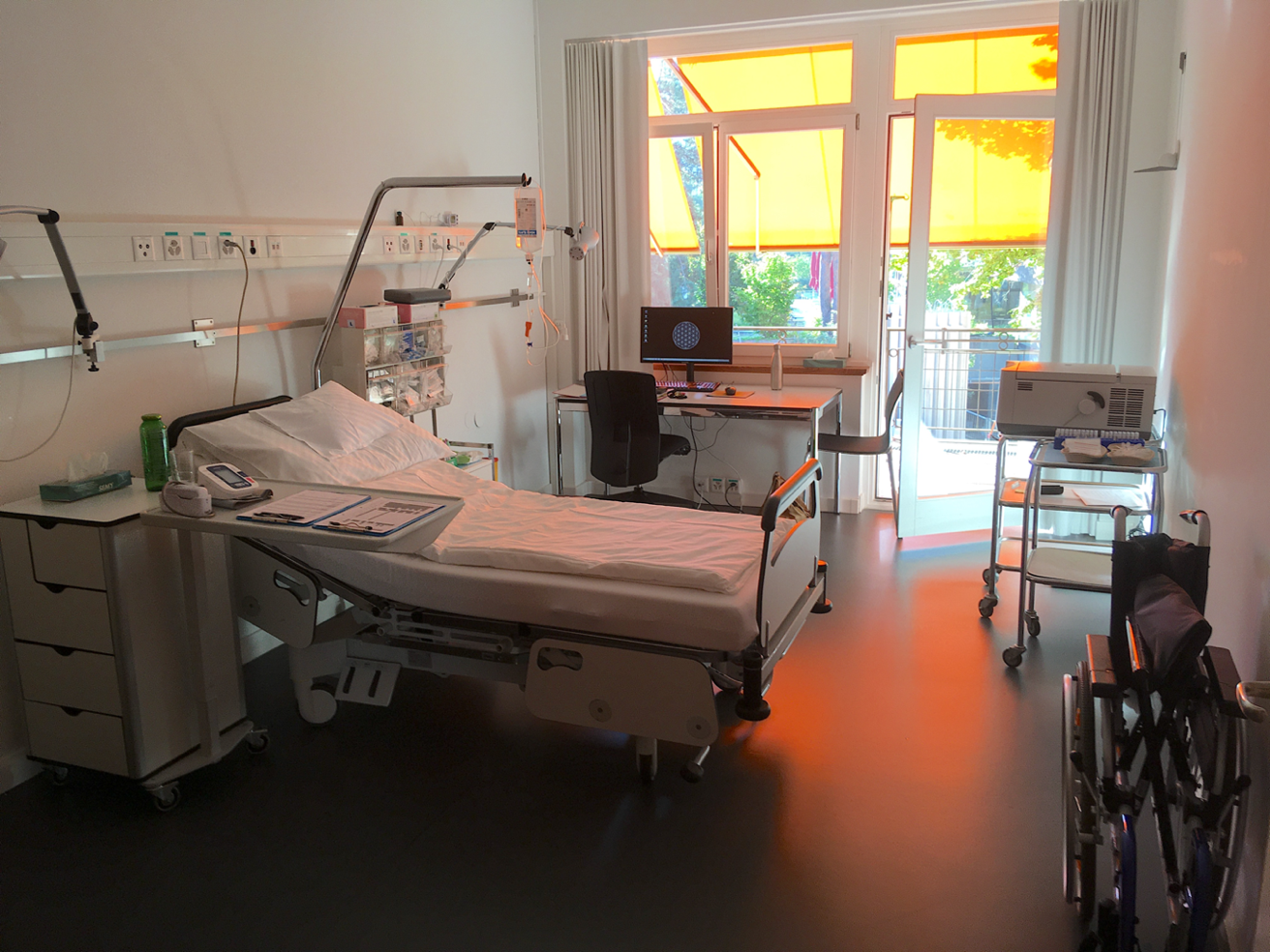
**

Supplementary Image S2: Hospital setting in which the studies were conducted


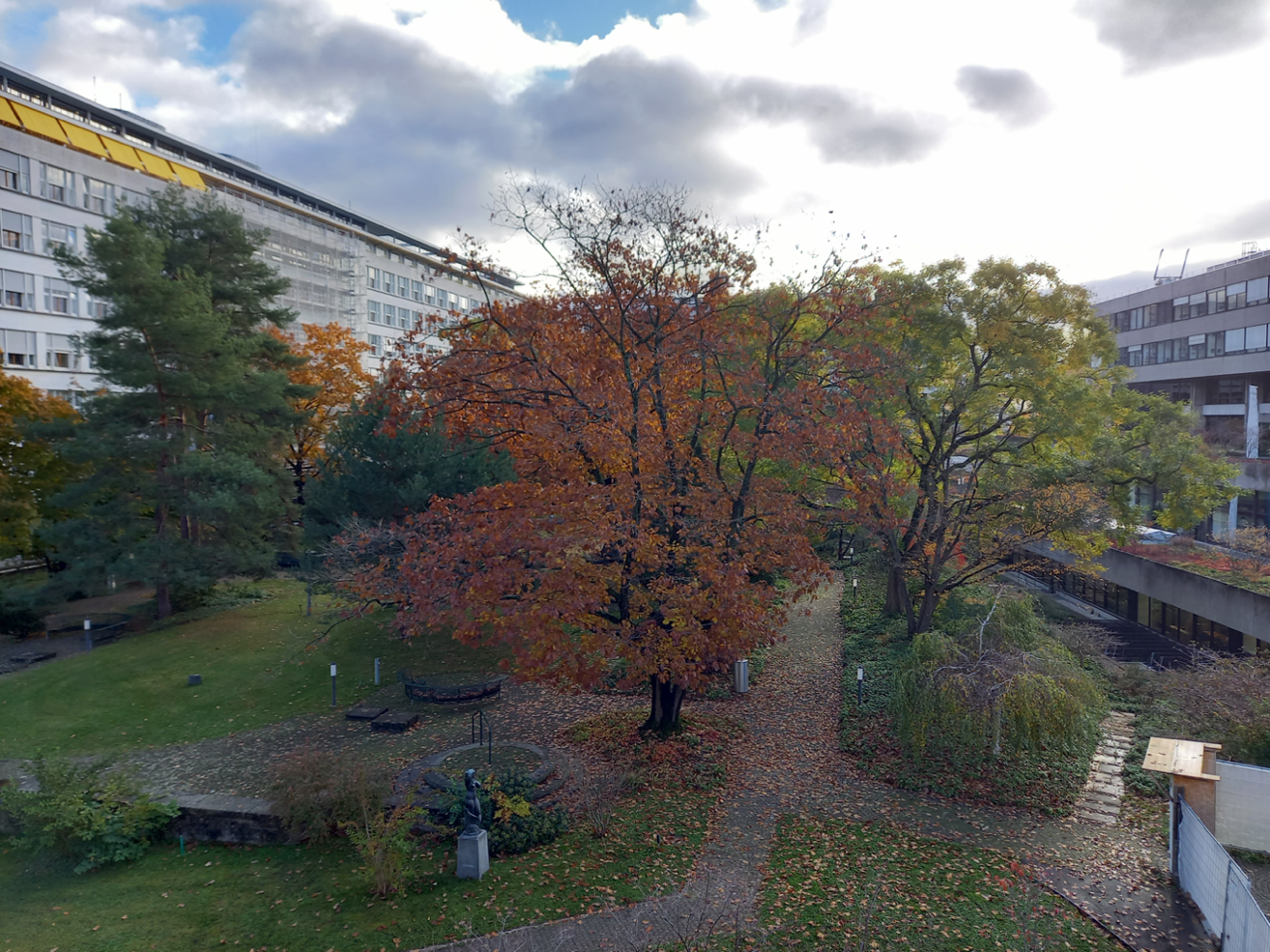


Supplementary Image S3: Balcony view of the hospital park


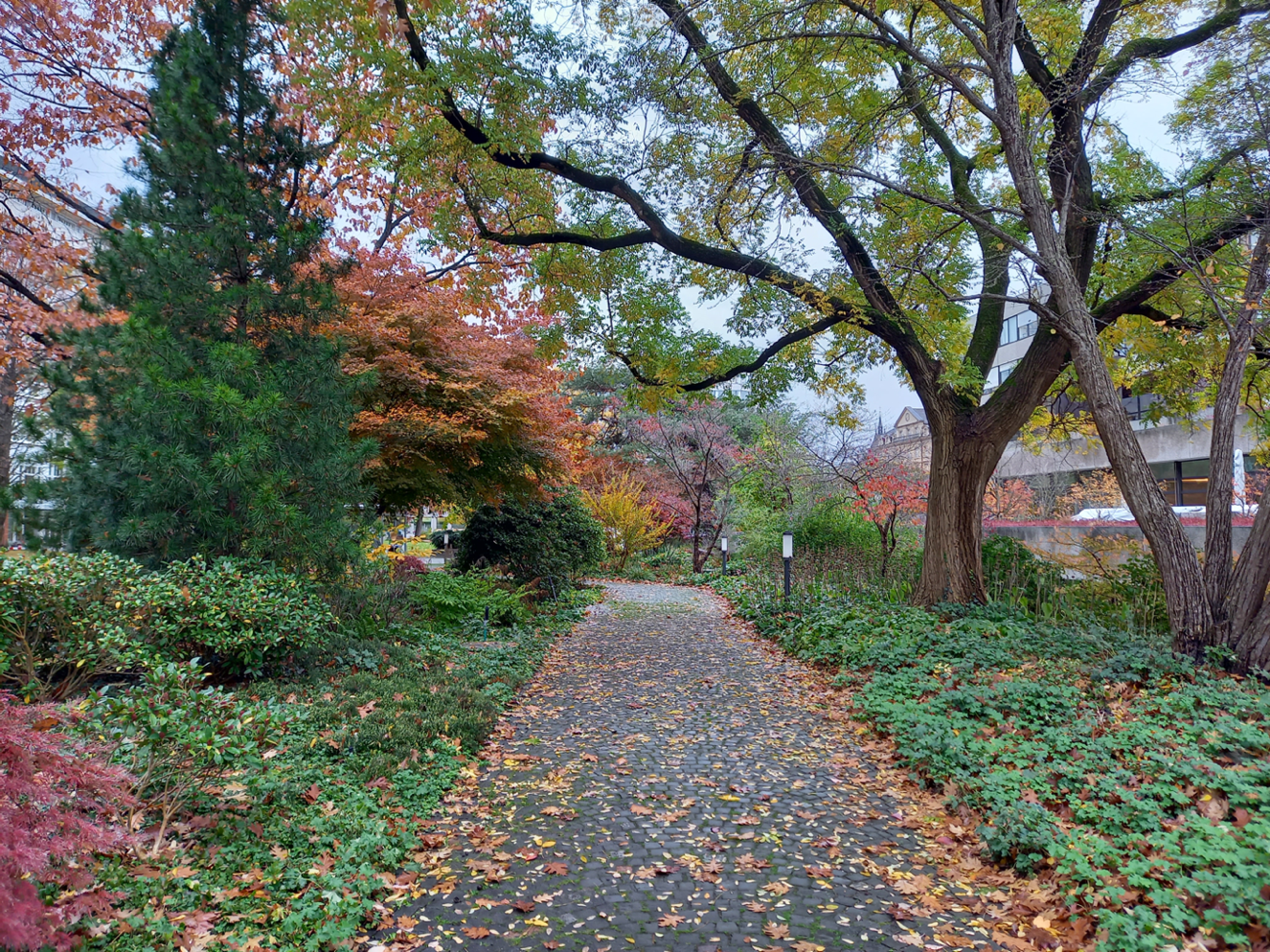


Supplementary Image S4: Hospital park

3

Ley *et al.:* Motivation and retrospective appraisal of psychedelic study participation

Ley *et al.:* Motivation and retrospective appraisal of psychedelic study participation

Ley *et al.:* Motivation and retrospective appraisal of psychedelic study participation

Ley *et al.:* Motivation and retrospective appraisal of psychedelic study participation
